# Editorial: Physiological Regulation and Homeostasis Among Coral Holobiont Partners

**DOI:** 10.3389/fphys.2022.921401

**Published:** 2022-05-10

**Authors:** Senjie Lin, Kefu Yu, Zhi Zhou

**Affiliations:** ^1^ Department of Marine Sciences, University of Connecticut, Groton, CT, United States; ^2^ School of Marine Sciences, Guangxi University, Nanning, China; ^3^ Department of Marine Sciences, College of Marine Sciences, Hainan University, Haikou, China

**Keywords:** coral holobiont, physiological regulation, homeostasis, heat stress, nutrient, microbiome, Symbiodiniaceae, bleaching

Scleractinian corals form the biological and physical framework for the most productive marine ecosystem in the oligotrophic oceans and play an important role in oceanographic processes (Costanza et al., 1997). The ecological success of coral reefs hinges on the symbiosis between the stony corals and the photosynthetic symbiodiniacean dinoflagellate (zooxanthellae), in which stony coral provides inorganic nutrients and carbon dioxide for the photosynthetic symbiont, whereas the symbionts supply the coral host with indispensable organic nutrients and oxygen (Shinzato et al., 2011). However, coral reefs, the home of the highest biodiversity in the ocean, are degrading rapidly in response to climate change and environmental pollution in the Anthropocene (Renegar and Riegl, 2005; Hughes et al., 2017; Serrano et al., 2018). Revealing how the symbiotic system adaptively responds to the variability of temperature and nutrients and maintain their homeostasis is necessary for efforts to rescue, restore, and protect the disappearing reefs in the face of climate change and environmental deterioration.

Coral lives in a complex holobiont composed of not only the cnidarian and zooxanthellae, but also the internal and external microbes (Rosenberg et al., 2007). For a long time, research on coral health and problems has laser focused on the mutualistic relationship between the cnidarian and the endosymbiotic symbiodiniacean dinoflagellates (zooxanthellae). With the advances of multi-omic technologies and physiological methodologies, a great deal has been learned about how the symbiotic relationship is started and maintained (Davy et al., 2012). A recent study indicated that the differential binding efficiency and specificity of coral lectins to the mannose moieties on the cell surface of different symbiodiniacean species might have significant effects on the success to initiate the symbiosis and maintain it during heat stress (Wang X et al., 2021). Nutrient compound exchanges are essential for the maintenance of a healthy and productive mutualism, and for the exchange, the compatibility of nutrient transport, assimilation, and storage machinery is vital (e. g., for translocation of glucose or glycerol, Lin et al., 2015), which might determine the symbiont preference (Lin et al., 2019). Environmental nutrients may also be important for the symbiodiniacean dinoflagellates to withstand heat stress (Zhou et al., 2021; Kirk et al. Nat Comm), but on the contrary nutrient overloading (eutrophication), particularly in combination with heat stress, can adversely impact the symbiosis and cause bleaching (D’Angelo and Wiedenmann, 2014; Lam et al., 2015; Humanes et al., 2016; Decarlo et al., 2020; Donovan et al., 2020). In addition, symbiont cell cycle modulation and host immunity are also critical for the maintenance of the symbiosis (Rivera and Davies, 2021).

In parallel to the continued effort to understand the coral-zooxanthella mutualism, there is a growing appreciation for the importance of bacteria in shaping the physiology and acclimatization of coral holobionts under novel and changing environments (Voolstra et al., 2021). In the last decade or so, an increasing number of studies examined the community structure and function of bacteria inside or on the surface of (mucus-associated) the host coral (e.g., Shnit-Orland and Kushmaro, 2009; Glasl et al., 2016). The need to study coral systems as holobionts is clear. Such effort has revealed that coral hosts, Symbiodiniaceae, and bacteria participate in the environmental adaptation of scleractinian coral (Yu et al., 2020a). For example, studies on scleractinian coral of the South China Sea have found that the trophic status (Xu et al., 2021), colony tissue thickness (Qin et al., 2020), Symbiodiniaceae density, Chl*a* content and tissue biomass (Qin et al., 2019), Symbiodiniaceae and bacteria diversity and community structure (Chen et al., 2019; Chen et al., 2021) of scleractinian corals are primarily driven by SST with latitudinal gradient shifts. These factors have important effects on coral holobionts tolerance; however, there are some differences between regions, coral species and even coral individuals (Yu et al., 2021a; Yu et al., 2021b; Yu et al., 2020b).

This Research Topic on *Physiological regulation and homeostasis among coral holobiont partners* aims to bring novel research findings together to bear on the molecular and physiological mechanisms in the partners of coral holobionts that underlie the establishment, maintenance and stability of symbiosis and adaptation to environmental changes. Nine exciting articles have been published.

One of the papers surveys the literature and draws perspectives about the importance of studying corals as holobionts using a holistic approach. Goulet et al. discussed the ramifications of how different genotypic combinations of the consortium constituents affected the coral holobiont entity. It was noted, using the zooxanthellae as the example, that the holobiont attributes might not necessarily be the sum of the constituents. The holobiont properties are consequential to holobiont fitness, which are in turn contributed by the myriad constituent organisms. The authors clearly articulated that a holistic approach requires evaluating the entire consortium instead of focusing on one species or component of the holobiont, investigating holobiont characteristics and performance, assessing parameters of as many of the holobiont participants as possible. The literature survey also revealed the paucity of data on the microbiome.

Four papers of the papers investigate the role of symbiotic microbiome in environmental adaptation of coral holobionts. Three papers focus on symbiotic Symbiodiniaceae. Wang et al. established monoclonal culture techniques and documented the physiological and ecological characteristics of Symbiodiniaceae C1 strain from the scleractinian coral *Galaxea fascicularis,* and this will be instrumental to further unraveling the roles of Symbiodiniaceae in the adaptive evolution of coral holobionts in future studies Wang et al. The work reported in Kirk et al. deals with transcriptional responses to trophic shifts in Symbiodiniaceae. The results showed that *Breviolum minutumstrain* SSB01 grew at a much faster rate and maintained stable photosynthetic efficiency when supplemented with organic nutrients compared to when only inorganic nutrients or no nutrient were provided. The authors concluded that these physiological changes were driven by massive transcriptomic changes involving DNA topoisomerases, histones, chromosome structural components, translation, ion transport, generation of second messengers, and phosphorylation. There was a high genetic diversity in Symbiodiniaceae with broad physiological plasticity within and between species, conferring great thermal tolerance.

Russnak et al. performed a comprehensive comparative experiment to explore the photosynthetic performance and tolerance as function of light and temperature of different genetic types of Symbiodiniaceae. The results clearly demonstrated that there were significant eco-physiological differences in light affinity and temperature tolerance among the tested species as well as strains within a species. The authors concluded that genetically different Symbiodiniaceae all had a broad photophysiological tolerance and substantial thermal plasticity. The effect of coral-associated bacterial communities on holobiont heat tolerance were examined by Connelly et al. The researchers investigated the microbial interactions in four *Pocillopora* coral colonies under heat stress and antibiotic treatment. The results showed that combined antibiotics and heat stress treatment significantly altered coral-associated bacterial communities and caused major changes in gene expression in both the coral and the symbiont *Cladocopium*. This study indicates the potential that the alteration of the bacterial community may contribute to or even exacerbate the stress imposed by heat. This paper provides further evidence that corals and their associated Symbiodiniaceae and bacteria communities engage in highly coordinated metabolic interactions that are crucial for coral holobiont health, homeostasis, and heat tolerance.

Three papers deal with nutrients and energy. One of these investigated the diversity of N_2_-fixing bacteria in the coral holobionts in the South China Sea (Liang et al.). Sixty-eight coral colonies were collected from six coral reef areas encompassing different environments ranging from 9°20′06″N to 22°34′55″N. PCR amplification and sequencing of *nifH* gene from the samples revealed a diverse diazotrophic flora dominated by Proteobacteria, Chlorobi, Cyanobacteria, and two unclassified phyla. Data further showed that coral-associated diazotrophs were common among coral demonstrate the potential importance of N_2_-fixing bacteria as the source of N-nutrient for corals, which live in the oligotrophic environment.

Lipids and fatty acids (FAs) are important constituents of cell and organelle membranes and have become a hot topic of research to understand the effects of eutrophication on corals. Liu et al. investigated the effects of nitrate (NO_3_^−^) enrichment on the respiration, photosynthesis, and FA compositions of *Pocillopora damicornis* larvae. Their survey showed that saturated FAs (SFAs) were the most abundant in *P. damicornis* larvae followed by polyunsaturated FAs (PUFAs) and monounsaturated FAs (MUFAs). Nitrate enrichment significantly changed the FA composition of *P. damicornis* larvae. The lipids of *P. damicornis* larvae became progressively saturated when the nitrate concentration was below 10 mM, due to the decreases of PUFAs and concomitant increases in SFAs. Such changes enabled the *P. damicornis* larvae to adapt to low-level nitrate enrichment (<10 mM). Moreover, with high nitrate levels, biomembrane restructuring in larvae may become ineffective, increasing respiration and rapidly consuming lipids, which could adversely affect the successful settlement and development of the larvae.

To understand how nutrient assimilation by the coral-Symbiodiniaceae entity is affected by heat stress, Tang et al. (2020) exposed *Pocillopora damicornis* to 32°C and measured the activity of ammonium assimilation enzymes among other physiological parameters. They found that the glutamine synthetase and glutamine oxoglutarate aminotransferase activities in the coral increased under heat stress, whereas that in the symbionts showed no significant changes. When the activities of glutamine synthetase were inhibited through glufosinate treatment, the heat stress response of the entity was intensified as indicated by the decline of symbiont density and chlorophyll content as well as the induction of coral antioxidant capacity and apoptosis. Therefore, ammonium assimilation contributes to the acclimatization of the coral-Symbiodiniaceae symbiotic association to high temperature.

While treatment experiments are powerful for controlling experimental conditions to test effects of one or few variables at a time, *in situ* studies provide understanding on the physiologies and behavior of the organisms in the natural complex environment. Xu et al. conducted a comparative study on corals in two distinct environments, the northern and southern sides of Weizhou Island in the South China Sea. They measured *in situ* electron transport rate (rETR), zooxanthella density and Chl*a* concentration, genetic diversity of the symbionts, stable isotopic signals of carbon, coral biomass, energy storage (in terms of carbohydrates), and superoxide dismutase (SOD) activity. ITS2 genotyping showed that *Galaxea fascicularis* contained either type C21 or D1a as the dominant symbiodiniacean symbiont depending on the regional environmental stress. Furthermore, the response of photosynthetic performance (the light use efficiency) and energy metabolism to the north-south environmental differences varied between species, and in the case of *P. verrucose* and *Montipora truncate*, the symbiont density (C1) in the north was higher, compensating for their lower photosynthetic efficiencies there, in comparison to the southern population.

The papers published in this special topic provide novel insights into how the coral holobionts respond to the environmental conditions, how the host, zooxanthellae, and bacteria may differentially contribute to the holobiont performance in the face of environmental stress. These findings demonstrate the value of *in situ* studies and treat the holobiont holistically in experiments. The *in situ* and holistic approach, coupled with omics and isotopic technologies, dealing with heat stress combined with different nutrient conditions, should be pursued much more in the future. Insights from such studies about how the coral holobionts respond and adapt to projected future climate and environment will be helpful for screening or breeding resilient genotypes in the urgently needed effort to rescue and restore the disappearing coral reefs, the treasure of the ocean. Finally, the Topic Editors would like to sincerely thank all the authors for their valuable contributions and all the reviewers for their efforts.
